# Supplementary material for: Mac-/Lactosylceramide regulates intestinal homeostasis and secretory cell fate commitment by facilitating Notch signaling
Source: eLife. 2025 Dec 23;14:RP106184. doi: 10.7554/eLife.106184 (PMC12726828; doi:10.7554/eLife.106184)
Supplement: Supplementary file 1. [file elife-106184-supp1.docx]

**Supplementary Table 1. Summary of tumor suppression genes identified in the screen.**

| **Gene/Label** | **Alleles identified** | **Function** | **Clone phenotype** |
| --- | --- | --- | --- |
| mam | 5 | Mastermind (mam) encodes a transcriptional coactivator that functions in the Notch signaling pathway. | ISC and EE tumor* |
| Gmer | 1 | Gmer exhibits GDP-L-fucose synthase activity that are essential for fucosylation of Notch. | ISC and EE tumor |
| O-fut1 | 2 | O-fut1 exhibits Notch binding activity and peptide-o-fucosyltransferase activity; positive regulation of Notch signaling pathway; and positive regulation of endocytosis. | ISC and EE tumor |
| GlcT (EA30, E230) | 2 | Glucosylceramide synthase (GIcT) encodes an enzyme that catalyzes the formation of glucosylceramide, the core component of glycosphingolipids. | ISC and EE tumor |
| n.d. (EA110) | 1 | n.d. | ISC and EE tumor |
| n.d. (EC230) | 1 | n.d. | ISC tumor** |

n.d., not determined; * the mutant clones containing excessive numbers of ISCs, undifferentiated cells, and EEs; ** the mutant clones containing mainly ISCs.
